# Initial Validation of Markerless Gait Analysis Using SPLYZA Motion: Waveform Agreement Under Practical Measurement Conditions

**DOI:** 10.3390/s26185809

**Published:** 2026-09-14

**Authors:** Yu Inoue, Ryo Yamasaki, Koji Ono, Shunsuke Yamashina, Kiyofumi Ando, Hiroyuki Doi

**Affiliations:** 1 School of Human Science, Kibi International University, Takahashi 716-8508, Japan; 2 Department of Rehabilitation, Kurashiki Heisei Hospital, Kurashiki 710-0826, Japan; ryo.yama.20.1@gmail.com; 3 Graduate School of Humanities and Social Sciences, Hiroshima University, Higashi-Hiroshima 739-8521, Japan; kojiono@hiroshima-u.ac.jp; 4 Faculty of Medicine, Shimane University, Izumo 693-8501, Japan; yamashina.s@med.shimane-u.ac.jp; 5 SPLYZA Inc., Hamamatsu 430-0805, Japan; ando.kiyofumi@splyza.com (K.A.); doi.hiroyuki@splyza.com (H.D.)

**Keywords:** markerless motion capture, gait analysis, waveform agreement, initial validation, kinematics

## Abstract

This study aimed to conduct an initial validation of markerless gait analysis using SPLYZA Motion by evaluating waveform agreement under practical measurement conditions. Joint angle waveforms were compared with those obtained from a marker-based motion capture system. SPLYZA Motion was found to be in good waveform agreement with the reference system for trunk and proximal lower-limb kinematics. Larger errors were observed in distal joints, particularly under occlusion and higher cadence; however, these differences were phase-dependent and did not substantially affect overall waveform patterns. These findings suggest that SPLYZA Motion may serve as a complementary tool for repeated waveform-based gait assessments under practical measurement conditions. Further validation in larger and more diverse populations is required before broader clinical application can be considered.

## 1. Introduction

The evaluation of gait characteristics plays a central role in understanding an individual’s physical condition and is widely used in rehabilitation and healthcare settings. Gait assessment commonly incorporates spatiotemporal parameters, such as walking speed and stride length [1,2,3], as well as kinematic parameters describing joint motion and segmental behavior [4,5]. Spatiotemporal parameters are particularly informative for evaluating mobility, fall risk, and overall functional status [1,2,3], while kinematic data provide detailed insight into movement strategies and joint-level abnormalities [4,5].

Gait kinematics are sensitive to age-related and pathological changes, making them important indicators of functional decline in older adults. Age-related and pathological alterations in gait kinematics are prevalent in older populations, with abnormal joint motion patterns frequently observed in individuals with neurological disorders or musculoskeletal conditions, such as osteoarthritis [6]. Moreover, recent studies have demonstrated the potential of kinematic gait parameters for the early detection of functional decline and prediction of disease onset [7,8], underscoring the importance of accurate and comprehensive gait analysis.

Despite their importance, an accurate assessment of gait kinematics is not always feasible in routine clinical practice. Optical motion capture systems are widely regarded as the gold standard for acquiring gait kinematics [9,10]; however, their high cost, complex setup, and time-consuming measurement procedures limit their accessibility in routine clinical practices. Despite the recognized clinical demand, only a limited proportion of patients can undergo laboratory-based gait assessments [11]. These limitations have motivated the development of alternative gait analysis approaches that are more feasible in clinical and community settings than traditional approaches.

Markerless motion capture techniques have emerged as practical alternatives; however, important methodological challenges remain. In recent years, markerless motion capture techniques have been proposed as practical alternatives, including depth-sensor–based systems and video-based approaches [12,13,14,15]. Previous studies have reported acceptable levels of reliability and validity for such systems under controlled conditions [16,17,18,19]. However, important methodological challenges remain. One critical bottleneck in two-dimensional video-based gait analysis is segmental occlusion, in which the limb on the far side of the camera is partially or fully hidden by the contralateral limb, potentially compromising the kinematic estimation accuracy. In addition, the robustness of markerless gait analysis under realistic constraints, such as limited frame rates commonly encountered in standard video recordings and variations in walking speed, has not been sufficiently examined.

Furthermore, most previous validation studies have primarily focused on representative values or average kinematic measures, with limited attention to phase-dependent waveform agreement and within-subject consistency across repeated trials. These aspects are particularly relevant when markerless systems are intended for longitudinal monitoring or pre–post comparisons within individuals.

The present study addressed these gaps by conducting an initial validation of a markerless gait analysis system using SPLYZA Motion. A distinguishing feature of the present validation was its combined focus on phase-dependent waveform agreement and within-subject consistency under practical measurement constraints, aspects that have received limited attention in previous validation studies. As an initial step toward future validation in older adults and clinical populations, the present study was conducted in healthy adults under controlled measurement conditions to establish the fundamental agreement between the markerless and reference systems. Joint kinematics obtained from two-dimensional video recordings were compared with reference data acquired using an optical three-dimensional motion capture system. Through this validation framework, we aimed to provide initial evidence regarding the applicability of markerless gait analysis for repeated and individualized assessments.

## 2. Materials and Methods

### 2.1. Participants

Four healthy adult men participated in this study (mean ± SD: age, 38.0 ± 7.8 years; height, 1.77 ± 0.04 m; body mass, 78.0 ± 13.2 kg). Participants were excluded if they had any current musculoskeletal injury, pain, or neurological condition that could affect gait performance.

All participants provided written informed consent prior to participation. The study protocol was approved by the Ethics Committee of Hiroshima University Graduate School of Humanities and Social Sciences (No. HR-ES-001489) and the Ethics Committee of Kibi International University (No. 23-33).

### 2.2. Experimental Design

This study was designed as an initial validation to examine the performance of a markerless gait analysis system under practical measurement conditions, including video recording at 30 frames per second (fps) and the presence of segmental occlusion inherent to the two-dimensional analysis.

Participants performed straight-line walking along a 5 m walkway under two controlled cadence conditions (80 and 100 steps/min), regulated using a metronome. These cadence conditions were selected to represent comfortable and slightly faster walking, thereby allowing the robustness of waveform agreement to be evaluated under clinically relevant walking conditions. To assess within-subject consistency, each participant completed 20 gait trials under each condition.

### 2.3. Data Acquisition

All gait trials were conducted indoors on a straight 5 m walkway. Video recordings were obtained using two iPhone SE (2nd generation) devices (Apple Inc., Cupertino, CA, USA) at a sampling rate of 30 fps.

One camera was positioned in front of the participant to capture the frontal plane motion, whereas the second camera was placed on the participant’s right side to record the sagittal plane motion. Both cameras were set at a height of 1.0 m from the floor and aligned parallel to the walking surface and positioned 5.0 m from the center of the walking path. The camera positions were adjusted to ensure that the participant’s full body was captured throughout the walking task. Data collection commenced after the participants achieved a stable walking rhythm at each prescribed cadence.

### 2.4. Motion Capture Systems

Three-dimensional kinematic data were acquired using an optical marker-based motion capture system (Vicon MX, Vicon Motion Systems, Oxford, UK), which served as the reference system. The system consisted of 10 infrared cameras sampling at 100 Hz and was calibrated according to the manufacturer’s standard procedures, prior to data collection. Reflective markers (14 mm diameter) were attached to anatomical landmarks based on a conventional full-body marker set compatible with the Plug-in Gait model, and marker trajectories were reconstructed in three-dimensional space using the system’s proprietary software.

Markerless gait analysis was performed using SPLYZA Motion (ver. 1.24.2; SPLYZA Inc., Shizuoka, Japan), which estimates the two-dimensional coordinates of anatomical landmarks from video recordings using a deep learning–based model. Skeletal coordinates were extracted for 27 landmarks representing the head, trunk, upper limbs, and lower limbs. For gait analysis, landmarks corresponding to the head, shoulders, elbows, hips, knees, ankles, heels, and toes were used.

**Figure 1:**
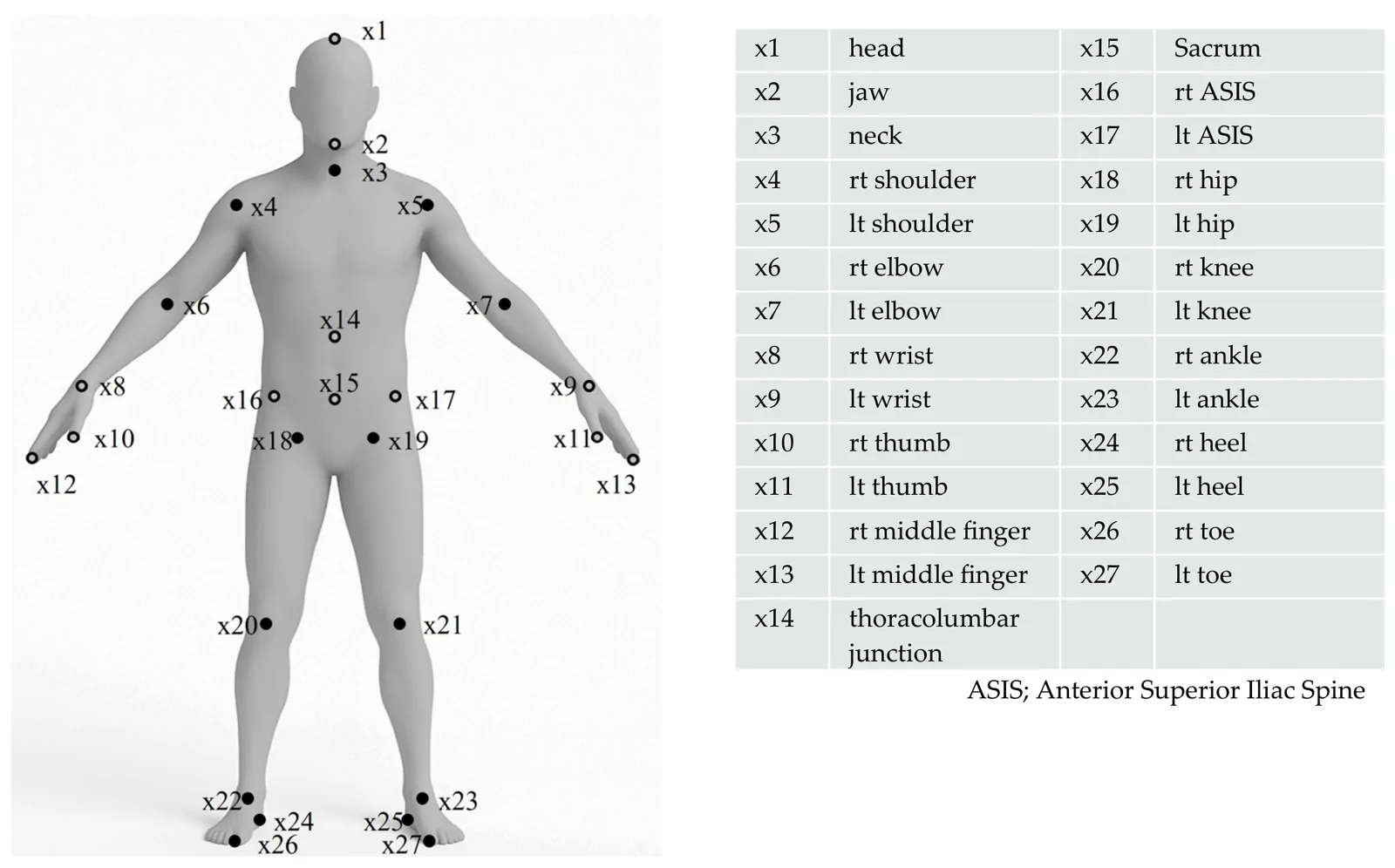
Skeletal landmark configuration. Filled circles indicate landmarks used for joint angle computation, whereas open circles indicate landmarks detected by SPLYZA Motion but not used in the present analysis.

Landmark data were obtained separately from recordings obtained in the frontal and sagittal planes, enabling the analysis of joint motion in both planes. Due to the two-dimensional nature of the recordings, partial occlusion of body segments on the far side of the camera was unavoidable in the sagittal plane view. A schematic illustration of the landmark configuration is presented in Figure 1. All video data were processed locally using the SPLYZA Motion framework without additional post-processing. A marker-based system was employed to provide high-resolution three-dimensional kinematic reference data for comparison with two-dimensional markerless gait analysis. All subsequent comparisons were conducted at the level of time-normalized gait cycles to ensure consistency between the reference and markerless systems.

### 2.5. Data Processing

Gait data obtained from the marker-based and markerless systems were processed using a consistent analytical framework to enable direct comparison. All analyses were performed on time-normalized gait cycles defined using identical criteria across systems.

For the marker-based system, three-dimensional marker trajectories were processed using the Plug-in Gait model. Two-dimensional skeletal coordinates extracted using SPLYZA Motion were exported and processed using custom scripts in MATLAB (MathWorks, Natick, MA, USA). For both systems, trajectory data were low-pass filtered using a second-order Butterworth filter with a cutoff frequency of 6 Hz, consistent with commonly adopted procedures in gait kinematic analyses [20,21]. Joint angles were calculated using custom MATLAB scripts. Hip, knee, ankle, and shoulder joint angles were calculated from the angles formed by three corresponding right-side anatomical landmarks. Trunk forward tilt was defined as the angle between the line connecting the neck and hip landmarks and the vertical axis, consistent with previous studies [16,17]. 

For each trial, a single steady-state gait cycle was extracted for subsequent analysis. For both systems, right initial contact (IC) was identified as the time point at which the relative horizontal distance between the ankle and hip landmarks reached its maximum. A gait cycle was defined between consecutive right IC events and resampled to 200 time-normalized data points (0–100% of the gait cycle) using spline interpolation. Each normalized gait cycle was subsequently divided into ten consecutive 10% intervals, each comprising 20 normalized data points, for phase-wise waveform analysis.

Frontal and sagittal plane recordings were synchronized using a digital visual counter recorded at the start of each trial. The counter displayed elapsed time with a resolution of 0.01 s and ten sequential visual indicators, allowing visual synchronization of the two recordings. The analysis intervals were defined based on the identified IC events to ensure consistency across the planes. 

Due to segmental occlusion in the sagittal plane recordings, temporary missing values were observed in the landmarks on the far side of the body. Landmark coordinates were generally estimated during partial occlusion; however, when occlusion exceeded the level at which the system was able to output landmark coordinates, coordinate values became temporarily unavailable. Missing data were imputed using the last observed value immediately preceding the missing interval. This approach was adopted to avoid generating artificial trajectories during intervals in which SPLYZA Motion produced no landmark estimates. This imputation procedure was consistently applied across all trials without manual correction.

### 2.6. Outcome Measures

The agreement between the marker-based and markerless systems was evaluated for joint kinematics under each cadence condition. 

Joint angle waveform agreement within each phase interval was quantified using the root mean square error (RMSE) and coefficient of multiple correlation (CMC). Phase-wise analyses were performed to characterize localized agreement across the gait cycle. 

The RMSE was calculated as the root mean square of the point-by-point differences between the joint angle waveforms obtained from the two systems over each phase interval. The RMSE values were normalized to the joint-specific reference ranges of motion and expressed as percentages (%RMSE) to allow comparison across joints and movement planes. Reference ranges of motion were defined according to the standardized joint range-of-motion measurement guidelines [22]. As no universally accepted threshold exists for %RMSE, the values were interpreted relative to the joint-specific magnitude and phase-dependent variation. 

CMC was used to assess joint angle waveform similarity within each phase interval, with values ≥ 0.75 interpreted as indicating good agreement [23,24]. 

All calculations were performed using MATLAB R2023b (MathWorks, Natick, MA, USA).

## 3. Results

### 3.1. Missing Landmark Data

Missing data rates for the landmark coordinates used to calculate each joint angle are presented in Table 1. In the sagittal plane, missing data were predominantly observed for shoulder flexion/extension on the occluded side, with mean missing rates of 59.2% and 63.6% at 80 and 100 steps/min, respectively. Missing data were also observed for ankle dorsiflexion/plantarflexion on the occluded side, with mean rates of 2.9% and 4.7%, respectively. In contrast, missing data rates were low for the other joint angles on both the non-occluded and occluded sides, as well as for joint angles in the frontal plane.

### 3.2. Joint Angle Waveforms

**Figure 2:**
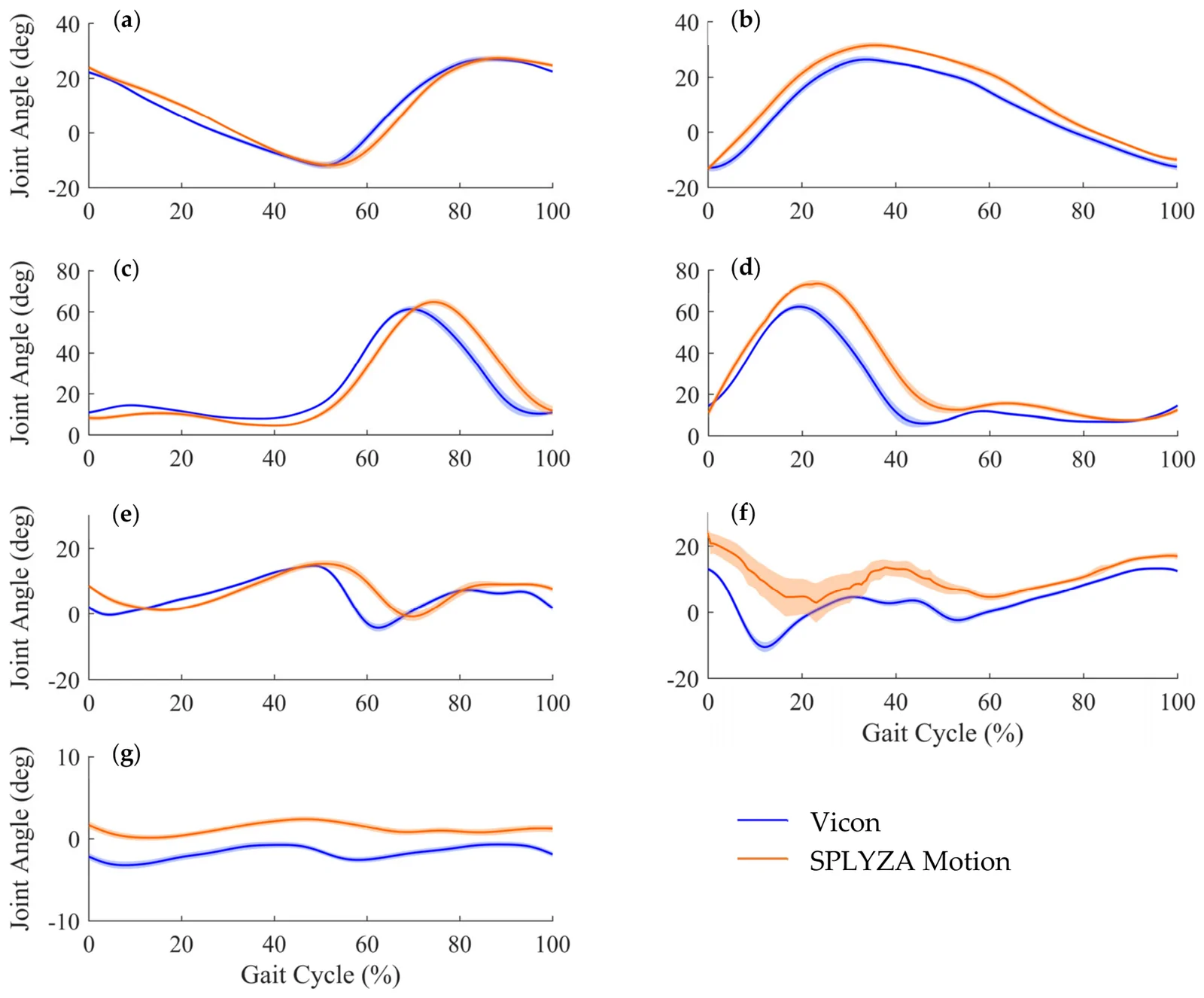
Mean sagittal-plane joint angle waveforms at 80 steps/min. (**a**) Rt hip flexion/extension; (**b**) Lt hip flexion/extension; (**c**) Rt knee flexion/extension; (**d**) Lt knee flexion/extension; (**e**) Rt ankle dorsi-flexion/plantar-flexion; (**f**) Lt ankle dorsi-flexion/plantar-flexion; (**g**) Trunk flexion/extension. Solid lines indicate the mean across all participants and all trials, and shaded areas represent the corresponding 95% confidence intervals.

**Figure 3:**
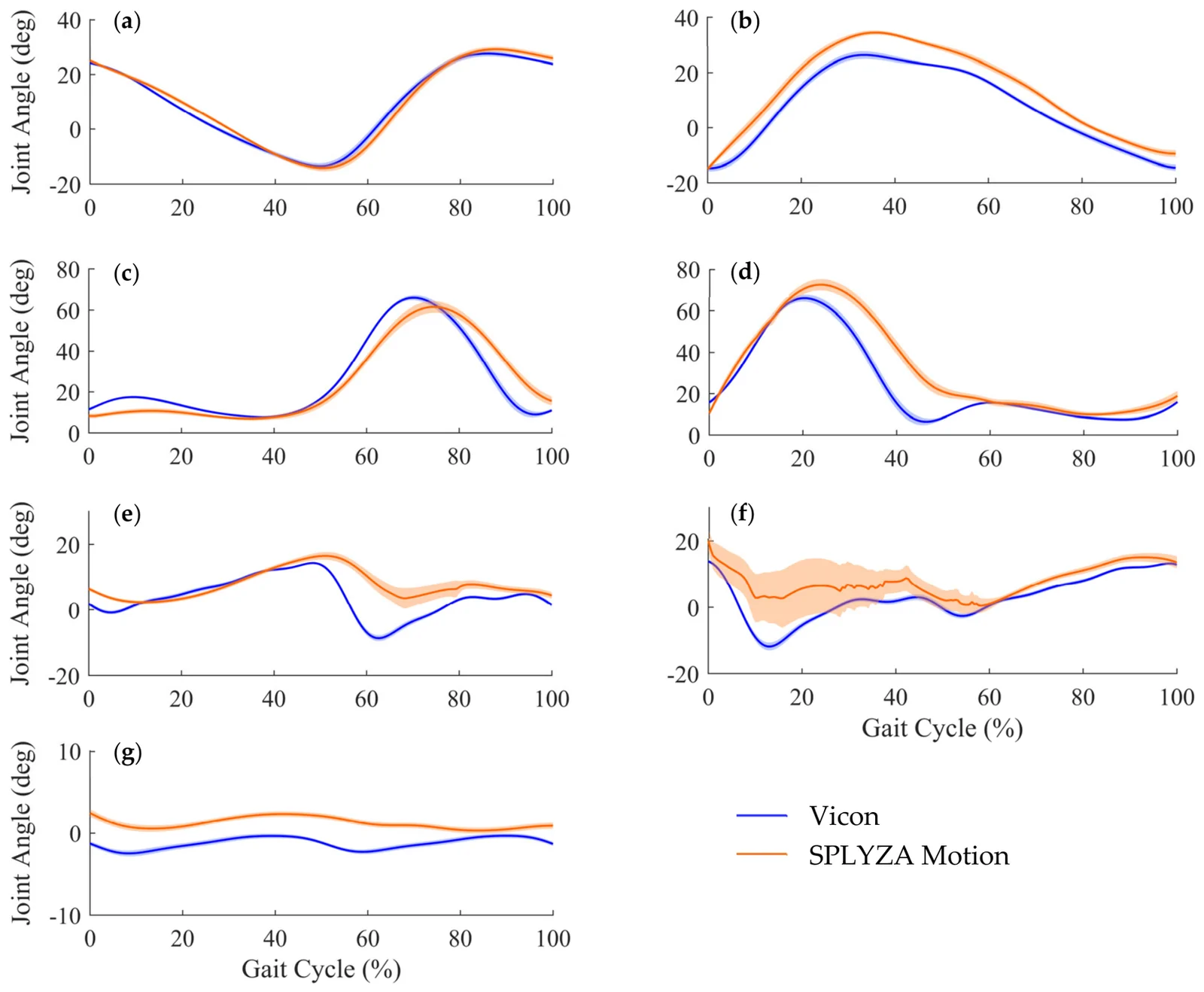
Mean sagittal-plane joint angle waveforms at 100 steps/min. (**a**) Rt hip flexion/extension; (**b**) Lt hip flexion/extension; (**c**) Rt knee flexion/extension; (**d**) Lt knee flexion/extension; (**e**) Rt ankle dorsi-flexion/plantar-flexion; (**f**) Lt ankle dorsi-flexion/plantar-flexion; (**g**) Trunk flexion/extension. Solid lines indicate the mean across all participants and all trials, and shaded areas represent the corresponding 95% confidence intervals.

The representative sagittal-plane joint angle waveforms are presented for selected joints to illustrate the principal waveform characteristics. Quantitative agreement analyses, however, were performed for all analyzed joints. Figure 2 and Figure 3 show the sagittal-plane joint angle waveforms obtained from the marker-based and markerless systems. Overall, the joint angle waveforms obtained from the markerless system closely followed those from the marker-based system for the proximal joints, including the hip and knee. In contrast, larger discrepancies were observed in distal joints, particularly at the ankle, where deviations in waveform shape and amplitude were more apparent.

### 3.3. Phase-Dependent %RMSE

**Figure 4:**
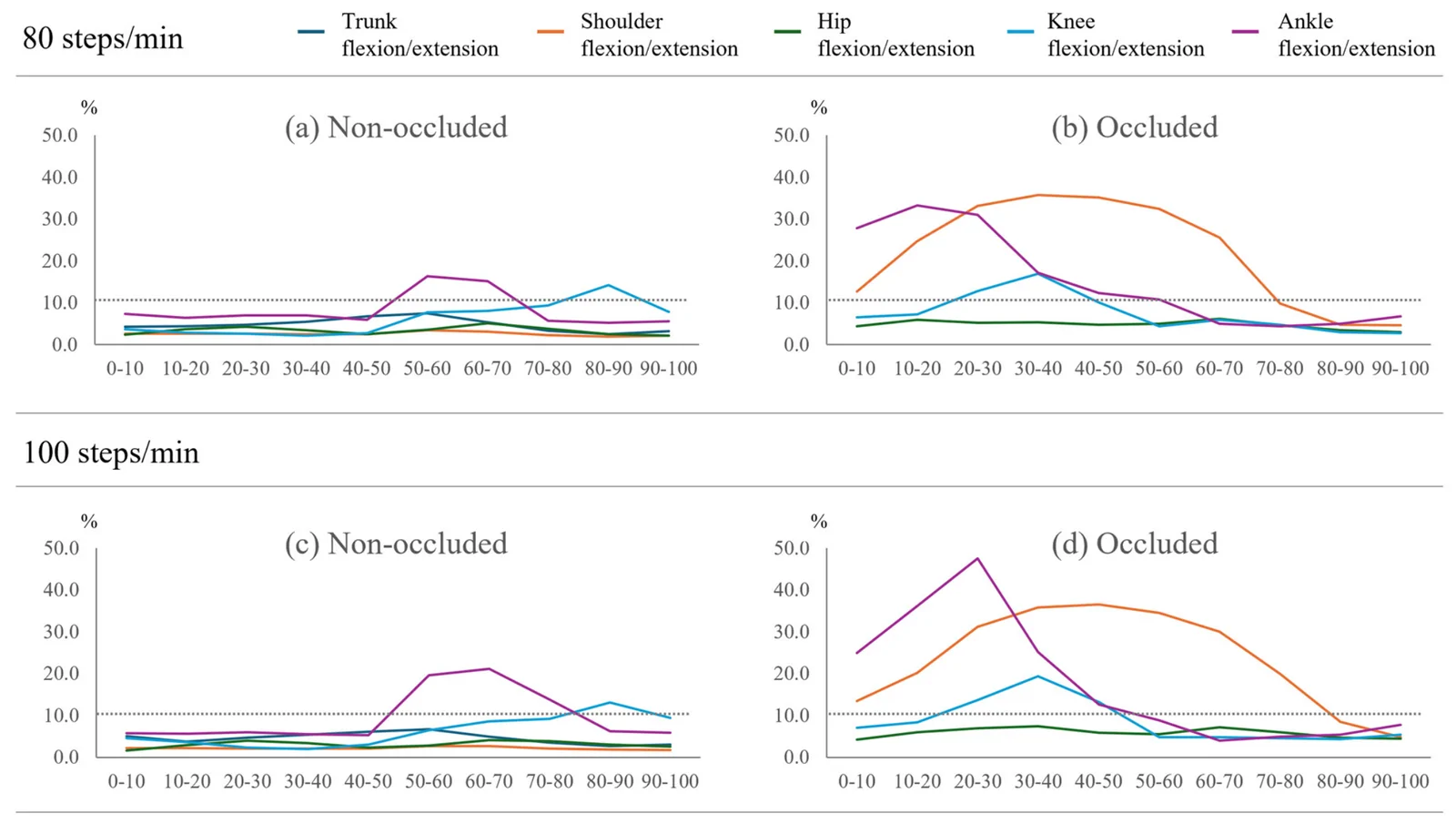
Phase-dependent %RMSE in the sagittal plane by cadence and occlusion condition. (**a**,**c**) Non-occluded side; (**b**,**d**) occluded side. Cadences were 80 steps/min for the upper panels and 100 steps/min for the lower panels. The dashed line indicates a reference level of 10% RMSE. Notable phase-specific increases in %RMSE were observed particularly for the ankle and for the shoulder on the occluded side.

**Figure 5:**
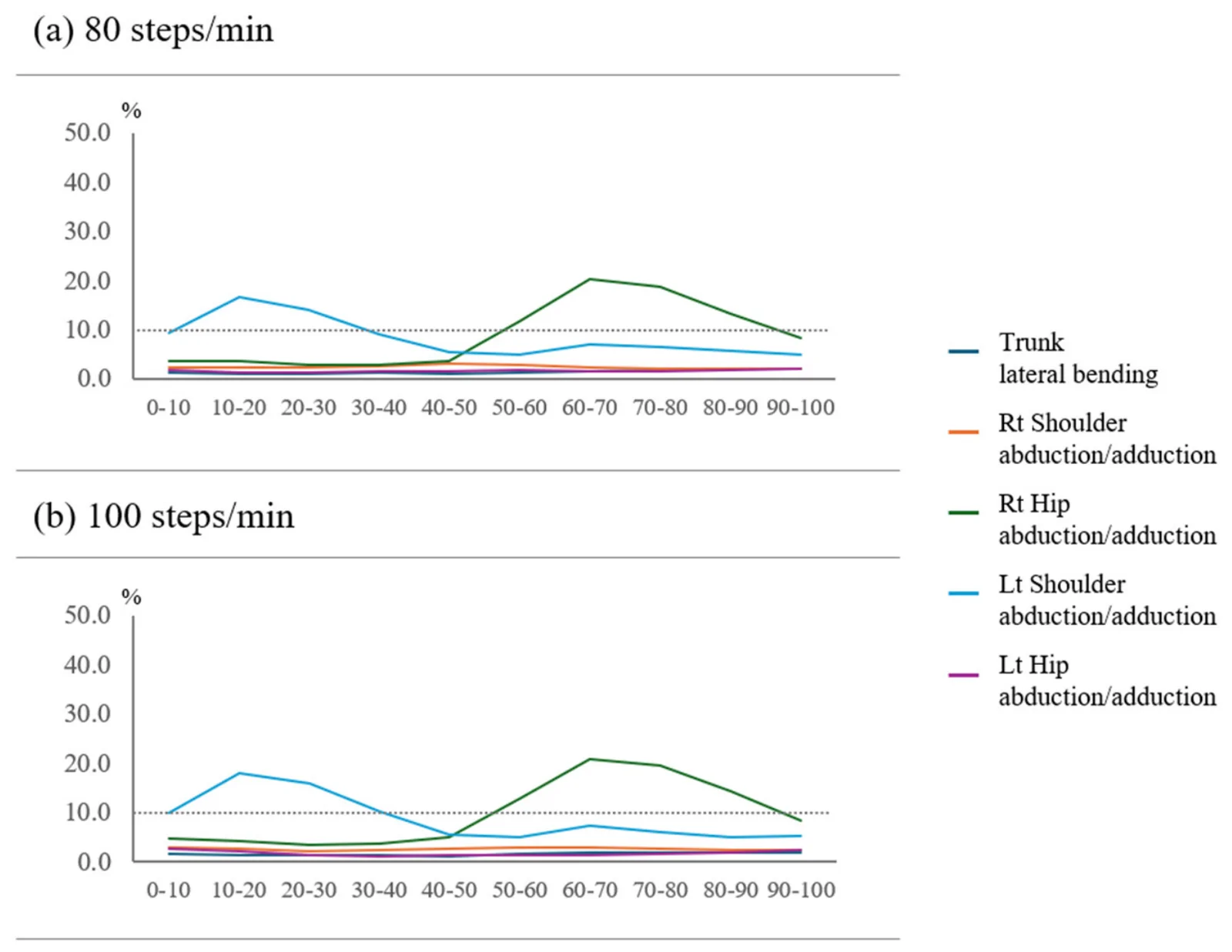
Phase-dependent %RMSE in the frontal plane by cadence and occlusion condition. (**a**) 80 steps/min; (**b**) 100 steps/min. The dashed line indicates a reference level of 10% RMSE. %RMSE values remained relatively stable across phases, with less phase-dependent variation than in the sagittal plane.

The phase-dependent %RMSE values in the sagittal and frontal planes are presented in Figure 4 and Figure 5, respectively. Corresponding numerical values for the 80 and 100 steps/min conditions are provided in Table 2 and Table 3. In the sagittal plane, %RMSE demonstrated clear phase-dependent variation across the gait cycle. Increases in %RMSE were observed during specific phases, particularly in distal joints, whereas values were generally lower in proximal joints, except for the shoulder on the occluded side. In contrast, %RMSE values in the frontal plane remained relatively stable, with minimal phase-dependent fluctuations.

Across cadence conditions, %RMSE values were generally higher at 100 steps/min than at 80 steps/min, although phase-dependent patterns were preserved.

### 3.4. Phase-Dependent CMC

**Figure 6:**
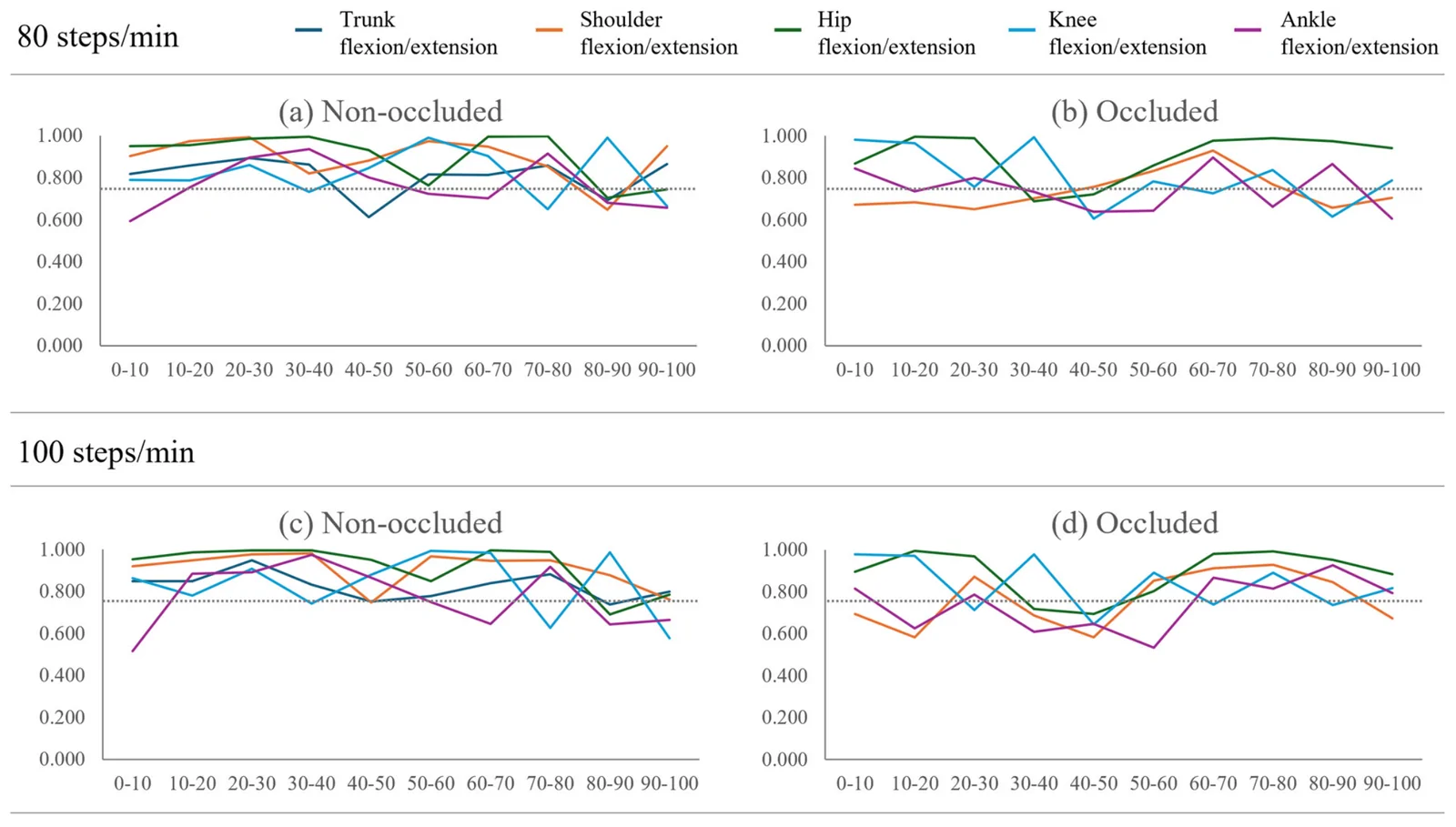
Phase-dependent CMC in the sagittal plane by cadence and occlusion condition. (**a**,**c**) Non-occluded side; (**b**,**d**) occluded side. Cadences were 80 steps/min for the upper panels and 100 steps/min for the lower panels. The dashed line indicates the reference threshold of 0.75 for CMC. Phase-specific reductions in CMC were observed particularly for the ankle and for the shoulder on the occluded side.

**Figure 7:**
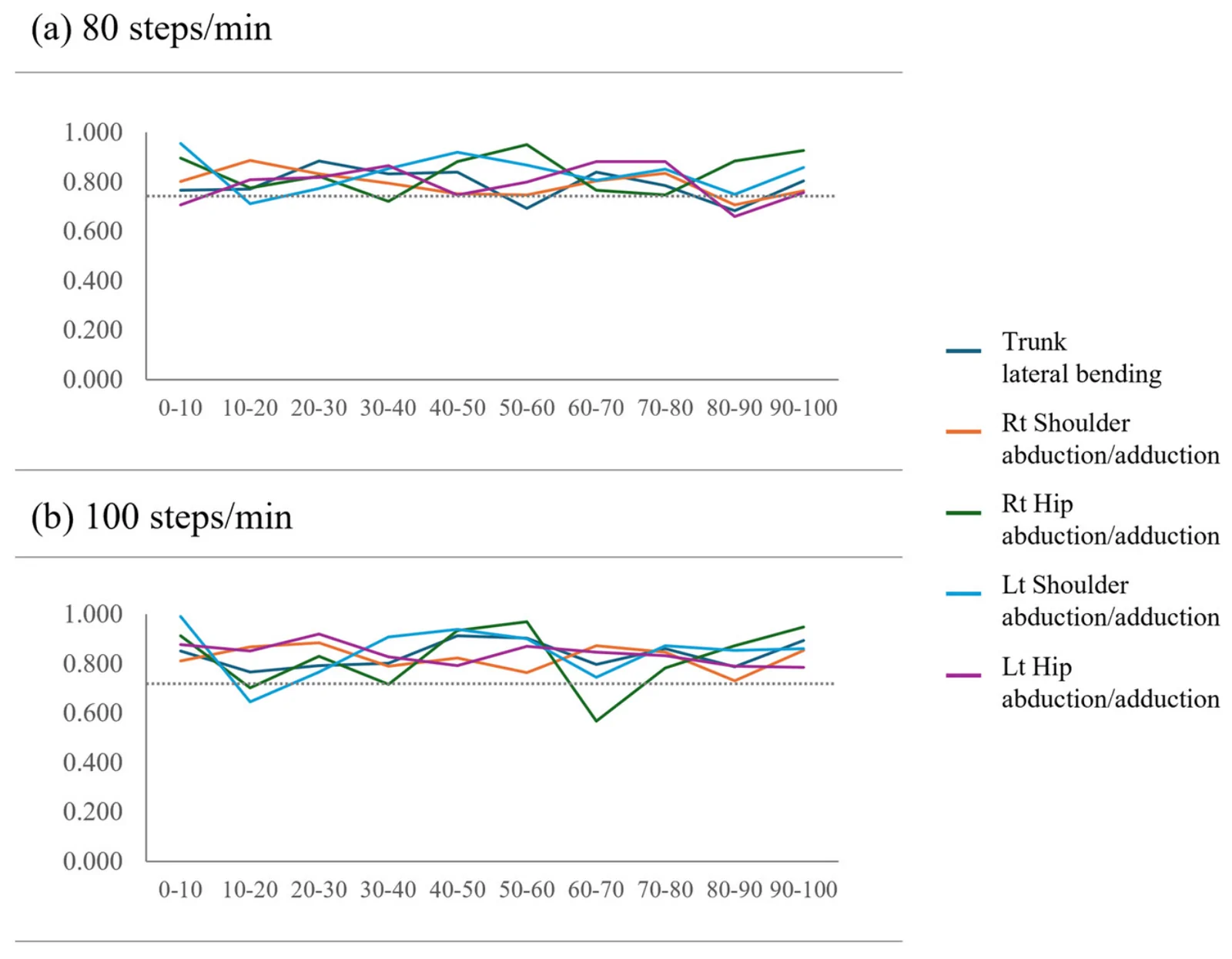
Phase-dependent CMC in the frontal plane by cadence and occlusion condition. (**a**) 80 steps/min; (**b**) 100 steps/min. The dashed line indicates the reference threshold of 0.75 for CMC. CMC values remained relatively stable across phases, with limited phase-dependent variation.

The phase-dependent CMC values in the sagittal and frontal planes are shown in Figure 6 and Figure 7, respectively. Detailed numerical results for the 80 and 100 steps/min conditions are reported in Table 4 and Table 5.

Overall, CMC values indicated good waveform agreement across most joints and phases. High levels of agreement were consistently observed in the proximal joints, whereas several phase-specific CMC values below 0.75 were observed, particularly for the occluded shoulder and ankle.

Reductions in CMC were observed in specific phases of the gait cycle in the sagittal plane, whereas CMC values in the frontal plane remained relatively stable with limited phase-dependent variation. Cadence had a modest effect on CMC, with slightly lower values at 100 steps/min compared with 80 steps/min.

### 3.5. Subject Variability

Within-subject variability of %RMSE and CMC is summarized in Table 6. Across participants, variability was generally small for both %RMSE and CMC, indicating consistent measurements across repeated trials within individuals.

## 4. Discussion

In this study, we evaluated the agreement between joint angle waveforms obtained using SPLYZA Motion and a three-dimensional motion capture system under practical measurement conditions. Missing landmark data were particularly frequent for shoulder flexion/extension on the occluded side and, to a lesser extent, for ankle dorsiflexion/plantarflexion on the occluded side, whereas missing data rates were low for most other joint-angle calculations. Proximal joints, including the trunk, hip, and knee, showed relatively good agreement with the reference system, whereas larger discrepancies were observed in distal joints and during phases affected by occlusion. These discrepancies were not uniformly distributed across the gait cycle but were localized to specific phases. In addition, waveform similarity was generally preserved, even under conditions in which absolute differences were present, and within-subject variability across repeated trials remained small. These findings suggest that markerless gait analysis may provide a certain level of kinematic information under practical conditions while also highlighting limitations related to joint location and occlusion.

The present study employed video recordings at 30 fps to reflect practical measurement conditions. Although lower temporal resolution may reduce the precision of rapid movement capture, waveform agreement was generally maintained under these conditions. The two cadence conditions enabled waveform agreement to be evaluated under both comfortable and slightly faster walking conditions. Slightly larger errors were observed at 100 steps/min, suggesting that temporal resolution limitations may have influenced estimation accuracy during faster movements. Accordingly, the present findings should be interpreted within the cadence range examined in this study, and further validation is warranted to determine the applicability of SPLYZA Motion under higher cadence conditions.

Occlusion influenced joint angle estimation in a phase-dependent rather than global manner. Missing landmark data were particularly frequent for shoulder flexion/extension on the occluded side and were also observed for ankle dorsiflexion/plantarflexion on the occluded side, whereas missing data rates were low for most other joint-angle calculations. In the sagittal plane, increased %RMSE and reduced CMC were primarily observed during specific phases, particularly in distal joints, whereas agreement remained relatively high outside these intervals. These findings suggest that the potential influence of missing landmark data and imputation using the last observed value differed across joint-angle calculations and may have been greater for the occluded-side shoulder and ankle. However, the extent to which this imputation influenced %RMSE and CMC could not be determined in the present study. In contrast, frontal plane measurements demonstrated comparatively stable agreement across phases, suggesting reduced occlusion effects. Similar condition-dependent error characteristics have been reported in previous markerless motion capture studies [25], indicating that such limitations may reflect the general characteristics of two-dimensional video-based analysis.

In the present study, waveform agreement was evaluated using %RMSE and CMC instead of discrete parameters, such as peak joint angles or range of motion. CMC is commonly used to assess waveform similarity [23], and values were generally high across conditions in the present study. In contrast, %RMSE varied across joints and phases, reflecting differences in absolute error magnitude. These findings suggest that temporal patterns of joint motion may be preserved even when absolute kinematic differences are present.

Within-subject variability remained small across repeated trials. By using repeated measurements rather than representative values alone, the present study evaluated measurement consistency under practical conditions. This characteristic may be particularly relevant for applications involving repeated assessments within individuals, such as pre–post comparisons and longitudinal monitoring.

Markerless motion capture approaches based on video analysis, including OpenPose, MediaPipe, and OpenCap, have been widely investigated. Previous studies have reported relatively good agreement for proximal joints but reduced accuracy for distal segments [12,13,26]. The present findings are generally consistent with these observations, particularly regarding increased errors in distal joints and under occlusion conditions [25]. The present validation is distinguished by its evaluation of waveform agreement and within-subject consistency under practical measurement conditions, including 30 fps video acquisition and segmental occlusion, rather than by introducing a new markerless gait analysis system. Therefore, these characteristics may represent the general limitations of two-dimensional markerless approaches rather than issues specific to SPLYZA Motion. These limitations arise primarily from perspective projection and self-occlusion, both of which are inherent to two-dimensional pose estimation and become particularly evident when body segments overlap in the sagittal plane [27,28]. Similar challenges have been recognized in other computer vision-based pose estimation applications, where the lack of depth information limits the robustness of two-dimensional approaches [27]. Recent advances in three-dimensional markerless pose estimation may help overcome these limitations by improving robustness against self-occlusion and perspective-related errors, representing an important direction for future clinical gait analysis [28]. SPLYZA Motion is designed to operate under practical measurement conditions without requiring specialized equipment or internet connectivity. These findings indicate that the characteristic strengths and limitations of two-dimensional markerless gait analysis are also evident when using the system under practical measurement conditions. These findings further suggest that SPLYZA Motion may serve as a complementary tool for gait analysis rather than as a direct substitute for laboratory-based motion capture systems. Such repeated waveform-based assessments may be useful for monitoring within-individual changes over time, for example during rehabilitation or longitudinal follow-up. However, further validation is required before broader clinical applicability can be established.

This study had several limitations. First, the sample size was small and limited to healthy adult males, restricting the generalizability of the findings to older adults and clinical populations with gait impairments. Second, the use of two-dimensional video recordings inherently introduced occlusion of body segments in the sagittal plane, which may have affected pose estimation accuracy. Missing landmark coordinates during these periods were handled using the last observed value rather than trajectory interpolation. Although this approach avoided reconstructing trajectories that were not estimated by SPLYZA Motion, it may also have influenced the waveform agreement observed during occluded phases. Missing data rates were low for most joint-angle calculations, whereas missing data were more frequent for shoulder flexion/extension and, to a lesser extent, ankle dorsiflexion/plantarflexion on the occluded side. Thus, the influence of this imputation approach may have been greater for these joint angles. Third, although synchronization was performed using a digital visual counter, synchronization accuracy between the frontal and sagittal plane recordings was not quantitatively verified. Therefore, a small synchronization error cannot be completely excluded and may have influenced waveform agreement. Finally, this study focused on waveform-based agreement and did not evaluate discrete kinematic parameters, such as peak joint angles. Future studies involving larger and more diverse populations, alternative approaches for handling missing landmark data, and three-dimensional markerless motion capture techniques are warranted to further establish the generalizability and clinical applicability of the present findings.

## 5. Conclusions

This initial validation demonstrated that SPLYZA Motion showed relatively good waveform agreement with a marker-based reference system for the trunk and proximal lower-limb joints under practical measurement conditions. Larger discrepancies were observed in distal joints and during higher cadence conditions; however, these differences were localized to specific phases of the gait cycle rather than being uniformly distributed across the waveform.

These findings suggest that SPLYZA Motion may serve as a complementary tool for repeated waveform-based gait assessments under practical measurement conditions. Further validation in larger and more diverse populations, including individuals with gait impairments, is required before broader clinical application can be considered.

## Figures and Tables

**Table 1:** Missing data in landmark coordinates used for joint angle calculations. Missing frames were defined as frames in which at least one X- or Y-coordinate required to calculate the corresponding joint angle was missing. Trials with missing data represent the number of trials containing at least one missing frame.

Joint	Motion	80 Steps/Min (N = 80)		100 Steps/Min (N = 80)	
		Missing Rate, % Mean (SD)	Trials with Missing Data, n	Missing Rate, % Mean (SD)	Trials with Missing Data, n
Sagittal plane					
Non-occluded					
Trunk	flexion/extension	0.2 (0.6)	6	0.4 (1.1)	10
Shoulder	flexion/extension	0.5 (2.5)	9	0.4 (1.1)	10
Hip	flexion/extension	0.1 (0.4)	2	0.1 (0.6)	4
Knee	flexion/extension	0.1 (0.4)	2	0.1 (0.6)	4
Ankle	dorsi-flexion/plantar-flexion	1.3 (10.7)	3	2.0 (10.9)	6
Occluded					
Shoulder	flexion/extension	59.2 (13.0)	80	63.6 (13.1)	80
Hip	flexion/extension	0.1 (0.4)	3	0.6 (5.1)	1
Knee	flexion/extension	0.4 (2.4)	4	0.6 (5.1)	1
Ankle	dorsi-flexion/plantar-flexion	2.9 (8.0)	18	4.7 (12.0)	33
Frontal plane					
Trunk	lateral bending	0.3 (1.0)	7	0.2 (1.1)	3
Rt Shoulder	abduction/adduction	0.3 (1.0)	8	0.2 (1.1)	3
Rt Hip	abduction/adduction	0.3 (1.0)	9	0.2 (1.1)	3
Lt Shoulder	abduction/adduction	0.2 (0.9)	4	0.2 (1.1)	3
Lt Hip	abduction/adduction	0.3 (1.2)	6	0.2 (1.1)	3

**Table 2:** Phase-dependent %RMSE of joint angles at 80 steps/min.

Joint	Motion	0–10	10–20	20–30	30–40	40–50	50–60	60–70	70–80	80–90	90–100	Mean
Sagittal plane												
Non-occluded												
Trunk	flexion/extension	4.2	4.4	4.7	5.5	6.8	7.5	5.3	3.3	2.4	3.2	4.7
Shoulder	flexion/extension	2.6	2.6	2.6	2.4	2.5	3.4	3.1	2.3	1.8	2.1	2.5
Hip	flexion/extension	2.4	3.6	4.3	3.4	2.5	3.5	5.1	3.8	2.5	2.1	3.3
Knee	flexion/extension	3.7	2.8	2.6	2.1	2.7	7.7	8.1	9.4	14.2	7.8	6.1
Ankle	dorsi-flexion/plantar-flexion	7.3	6.4	7.0	7.0	5.9	16.3	15.2	5.7	5.2	5.6	8.1
Occluded												
Shoulder	flexion/extension	12.6	24.7	33.2	35.7	35.1	32.5	25.6	9.9	4.7	4.6	21.9
Hip	flexion/extension	4.4	5.9	5.2	5.3	4.7	4.9	6.2	4.7	3.5	3.0	4.8
Knee	flexion/extension	6.6	7.2	12.8	17.0	10.1	4.4	6.0	4.7	3.0	2.7	7.4
Ankle	dorsi-flexion/plantar-flexion	27.7	33.2	30.9	17.1	12.3	10.8	4.9	4.4	5.0	6.7	15.3
Frontal plane												
Trunk	lateral bending	1.2	0.9	1.0	1.1	1.1	1.3	1.5	1.7	2.0	1.9	1.4
Rt Shoulder	abduction/adduction	2.3	2.2	2.2	2.6	2.9	2.8	2.3	2.1	2.0	2.1	2.4
Rt Hip	abduction/adduction	3.5	3.4	2.9	2.6	3.6	11.7	20.3	18.6	13.2	8.4	8.8
Lt Shoulder	abduction/adduction	9.2	16.6	14.1	8.9	5.4	4.9	7.0	6.4	5.5	4.8	8.3
Lt Hip	abduction/adduction	1.7	1.3	1.3	1.3	1.4	1.6	1.5	1.4	1.7	2.0	1.5

Values are expressed as percentages normalized to joint-specific reference ranges of motion.

**Table 3:** Phase-dependent %RMSE of joint angles at 100 steps/min.

Joint	Motion	0–10	10–20	20–30	30–40	40–50	50–60	60–70	70–80	80–90	90–100	Mean
Sagittal plane												
Non-occluded												
Trunk	flexion/extension	5.1	3.8	4.7	5.4	6.2	6.7	5.0	3.5	2.7	3.1	4.6
Shoulder	flexion/extension	2.2	2.2	2.1	2.0	2.1	2.7	2.7	2.1	1.8	1.8	2.2
Hip	flexion/extension	1.6	2.9	4.0	3.4	2.3	2.8	4.1	3.8	3.1	2.6	3.1
Knee	flexion/extension	4.6	3.7	2.3	2.0	3.1	6.4	8.6	9.2	13.1	9.5	6.2
Ankle	dorsi-flexion/plantar-flexion	5.7	5.7	6.0	5.5	5.3	19.6	21.1	13.8	6.3	5.9	9.5
Occluded												
Shoulder	flexion/extension	13.5	20.2	31.2	35.8	36.6	34.6	30.0	20.0	8.5	4.8	23.5
Hip	flexion/extension	4.3	6.0	7.0	7.4	5.9	5.6	7.2	6.0	4.8	4.5	5.9
Knee	flexion/extension	7.1	8.4	13.7	19.4	13.3	4.8	4.9	4.6	4.3	5.4	8.6
Ankle	dorsi-flexion/plantar-flexion	24.9	36.2	47.6	25.2	12.6	8.9	4.0	4.9	5.4	7.8	17.7
Frontal plane												
Trunk	lateral bending	1.4	1.3	1.2	1.2	1.0	1.4	1.6	1.6	1.7	1.7	1.4
Rt Shoulder	abduction/adduction	2.7	2.5	2.0	2.1	2.6	2.8	2.8	2.5	2.2	2.2	2.4
Rt Hip	abduction/adduction	4.7	4.0	3.4	3.6	4.9	12.6	20.8	19.5	14.3	8.1	9.6
Lt Shoulder	abduction/adduction	9.9	18.0	15.8	10.1	5.4	5.0	7.3	5.8	4.8	5.1	8.7
Lt Hip	abduction/adduction	2.4	1.9	1.1	1.0	1.2	1.3	1.3	1.4	1.8	2.2	1.6

Values are expressed as percentages normalized to joint-specific reference ranges of motion.

**Table 4:** Phase-dependent CMC of joint angles at 80 steps/min.

Joint	Motion	0–10	10–20	20–30	30–40	40–50	50–60	60–70	70–80	80–90	90–100	Mean
Sagittal plane												
Non-occluded												
Trunk	flexion/extension	0.816	0.857	0.894	0.861	0.612	0.815	0.812	0.859	0.692	0.865	0.808
Shoulder	flexion/extension	0.904	0.974	0.994	0.821	0.883	0.973	0.949	0.854	0.646	0.950	0.895
Hip	flexion/extension	0.950	0.954	0.986	0.995	0.932	0.763	0.994	0.996	0.704	0.745	0.902
Knee	flexion/extension	0.788	0.787	0.859	0.733	0.846	0.991	0.903	0.649	0.992	0.663	0.821
Ankle	dorsi-flexion/plantar-flexion	0.593	0.753	0.896	0.936	0.801	0.722	0.703	0.916	0.681	0.657	0.766
Occluded												
Shoulder	flexion/extension	0.671	0.684	0.650	0.703	0.756	0.833	0.929	0.769	0.658	0.705	0.736
Hip	flexion/extension	0.867	0.996	0.989	0.689	0.722	0.858	0.976	0.988	0.974	0.942	0.900
Knee	flexion/extension	0.981	0.966	0.757	0.993	0.605	0.783	0.726	0.838	0.616	0.788	0.805
Ankle	dorsi-flexion/plantar-flexion	0.844	0.736	0.800	0.733	0.639	0.644	0.896	0.662	0.866	0.606	0.743
Frontal plane												
Trunk	lateral bending	0.767	0.772	0.885	0.832	0.840	0.694	0.839	0.785	0.684	0.804	0.790
Rt Shoulder	abduction/adduction	0.802	0.887	0.833	0.795	0.752	0.748	0.804	0.834	0.708	0.764	0.793
Rt Hip	abduction/adduction	0.896	0.776	0.823	0.721	0.882	0.951	0.766	0.747	0.884	0.928	0.837
Lt Shoulder	abduction/adduction	0.955	0.712	0.773	0.854	0.921	0.868	0.806	0.851	0.749	0.858	0.834
Lt Hip	abduction/adduction	0.708	0.810	0.818	0.867	0.747	0.800	0.882	0.883	0.661	0.756	0.793

Values ≥ 0.75 indicate good waveform agreement.

**Table 5:** Phase-dependent CMC of joint angles at 100 steps/min.

Joint	Motion	0–10	10–20	20–30	30–40	40–50	50–60	60–70	70–80	80–90	90–100	Mean
Sagittal plane												
Non-occluded												
Trunk	flexion/extension	0.850	0.849	0.948	0.833	0.751	0.777	0.840	0.881	0.739	0.800	0.827
Shoulder	flexion/extension	0.920	0.947	0.976	0.981	0.746	0.966	0.945	0.948	0.877	0.762	0.907
Hip	flexion/extension	0.953	0.986	0.996	0.996	0.950	0.848	0.997	0.989	0.690	0.786	0.919
Knee	flexion/extension	0.864	0.781	0.907	0.743	0.880	0.994	0.983	0.627	0.986	0.578	0.834
Ankle	dorsi-flexion/plantar-flexion	0.515	0.885	0.893	0.974	0.867	0.749	0.646	0.917	0.644	0.663	0.775
Occluded												
Shoulder	flexion/extension	0.694	0.583	0.871	0.687	0.583	0.853	0.912	0.929	0.845	0.673	0.763
Hip	flexion/extension	0.896	0.995	0.968	0.717	0.694	0.803	0.979	0.992	0.953	0.884	0.888
Knee	flexion/extension	0.978	0.971	0.712	0.977	0.643	0.890	0.739	0.890	0.736	0.816	0.835
Ankle	dorsi-flexion/plantar-flexion	0.815	0.625	0.786	0.607	0.646	0.533	0.867	0.814	0.926	0.792	0.741
Frontal plane												
Trunk	lateral bending	0.852	0.767	0.793	0.803	0.914	0.903	0.798	0.861	0.787	0.894	0.837
Rt Shoulder	abduction/adduction	0.811	0.868	0.886	0.790	0.823	0.765	0.872	0.846	0.730	0.853	0.824
Rt Hip	abduction/adduction	0.913	0.703	0.830	0.717	0.934	0.970	0.568	0.783	0.872	0.948	0.824
Lt Shoulder	abduction/adduction	0.991	0.645	0.766	0.908	0.939	0.901	0.746	0.874	0.854	0.862	0.849
Lt Hip	abduction/adduction	0.878	0.850	0.920	0.828	0.792	0.871	0.846	0.833	0.790	0.785	0.839

Values ≥ 0.75 indicate good waveform agreement.

**Table 6:** Within-subject variability of %RMSE and CMC across repeated trials.

Joint	Motion	SD at 80 Steps/Min		SD at 100 Steps/Min	
		%RMSE, %	CMC	%RMSE, %	CMC
Sagittal plane					
Non-occluded					
Trunk	flexion/extension	1.2	0.202	1.7	0.190
Shoulder	flexion/extension	1.1	0.131	0.7	0.121
Hip	flexion/extension	1.5	0.100	1.7	0.097
Knee	flexion/extension	1.9	0.165	2.3	0.144
Ankle	dorsi-flexion/plantar-flexion	3.3	0.211	5.2	0.196
Occluded					
Shoulder	flexion/extension	3.9	0.226	3.6	0.196
Hip	flexion/extension	1.5	0.108	3.2	0.125
Knee	flexion/extension	2.6	0.175	4.9	0.166
Ankle	dorsi-flexion/plantar-flexion	7.1	0.231	10.2	0.223
Frontal plane					
Trunk	lateral bending	0.7	0.251	0.7	0.216
Rt Shoulder	abduction/adduction	0.5	0.243	0.5	0.219
Rt Hip	abduction/adduction	1.9	0.179	1.7	0.185
Lt Shoulder	abduction/adduction	0.5	0.234	0.5	0.214
Lt Hip	abduction/adduction	1.9	0.201	1.7	0.159

## Data Availability

The data presented in this study are available on request from the corresponding author due to ethical and privacy considerations.

## Abbreviations

The following abbreviations are used in this manuscript:

ASIS	Anterior Superior Iliac Spine
CMC	Coefficient of Multiple Correlation
fps	frames per second
IC	Initial Contact
RMSE	Root Mean Square Error
%RMSE	Percentage Root Mean Square Error
TO	Toe-Off
